# The Philosophy of Health; or, an Exposition of the Physical and Mental Constitution of Man, with a View to the Promotion of Human Longevity and Happiness

**Published:** 1838-04

**Authors:** 


### Art. V.

1. *The Philosophy of Health; or, an Exposition of the Physical and Mental Constitution of Man, with a View to the Promotion of Human Longevity and Happiness.* By Southwood Smith, m.d., Physician to the London Fever Hospital, &c. &c. *Volume Second.—London*, 1837. 8vo. pp. 448.
2. *The Philosophy of Living.* By Herbert Mayo, f.r.s., Senior Surgeon of the Middlesex Hospital.—*London*, 1837. 8vo. pp. 318.
3. *Management of the Organs of Digestion, in Health and Disease.* By Herbert Mayo, f.r.s., &c.—*London*, 1837. 8vo. pp. 198.
4. *The Economy of Health; or, the Stream of Human Life from the Cradle to the Grave: with Reflections, moral, physical, and philosophical, on the successive Phases of Human Existence, the Maladies to which they are subject, and the Dangers that may be averted.* By James Johnson, m.d., Physician Extraordinary to the King. —*London*, 1837. 8vo. pp. 229.
5. *A Popular Treatise on Diet and Regimen; intended as a Text-book for the Invalid and the Dyspeptic.* By W. H. Robertson, m.d.—*London*, 1835. 8vo. pp. 251.
6. *A Treatise on Diet; with a View to establish, on practical Grounds, a System of Rules for the Prevention and Cure of the Diseases incident to a disordered State of the Digestive Functions.* By J. A. Paris, m.d. &c. *Fifth Edition, corrected, enlarged, and nearly rewritten.—London*, 1837. 8vo. pp. 414.
7. *Observations on the Preservation of Health, in Infancy, Youth, Manhood, and Age; with the best Means of improving the moral and physical Condition of Man.* By John Harrison Curtis, Esq.—*London*, 1837. 8vo. pp. 128.
8. *Education Physique des Jeunes Filles; ou, Hygiène de la Femme avant le Mariage.* Par A. M. Bureaud-Riofrey, Docteur en Médecine de la Faculté de Paris, &c.—*Paris*, 1835. 8vo. pp. 352.

*Physical Education of Young Women, &c.* By A. M. Bureaud-Riofrey, m.d. of the Faculty of Paris, &c. &c.—*Paris*, 1835.

9. *The Philosophy of Living; or, the Way to enjoy Life and its Comforts.* By Caleb Ticknor, a.m. m.d.—*New York*, 1836. 8vo. pp. 334.

The above are the titles of but a portion of the numerous publications which have recently issued from the press for the instruction of general readers. However various in method and in merit, we see the announcement of all such with satisfaction, believing that the public are in some degree benefited by each. The world has readers of all sorts: some are attracted by a grave treatise; some are pleased with a popular view; some disregard higher recommendations, if the decencies, the foibles, and prejudices of the world be but respected; and some are infallibly caught by those who angle with a long title-page.

I. We derived so much gratification from Dr. Southwood Smith's first volume, that we waited almost impatiently for the second. Like the first, it is full of information, conveyed in very lucid language, although we think the subjects treated of in it are, many of them, such as will not impress cursory examiners with a true idea of its great interest. The running title-pages include many particulars from which idle readers may perhaps turn away; affrighted by *Calculations*, *General Results*, *Experiments*, *Development of Glands*, *Chemical Action*, *Ultimate Processes*, *Evidence of Absorption*, *Endosmose*, and many other terms indicative to the physiologist of the completeness of the range taken by the author, but less attractive to the superficial and selfish persons for whom authors, wiser in their generation, are careful to provide allurements. Associating habitually, we should conjecture, with a circle distinguished by a high degree of mental cultivation, and the preservation, by mutual example and influence, of great intellectual activity, Dr. Southwood Smith has written a work which is eminently adapted to the readers which such a circle may be expected to create, even to the youngest of such a circle. To furnish such readers with such information,—information which it is always most difficult for them to procure,—and in terms and in a manner adapted to their taste and acquirements, indicates an ambition of a much higher kind than animates popular writers on medicine in general.

The present volume of the "Philosophy of Health" comprehends the subjects of Respiration, Animal (and Vegetable) Heat, Digestion, Secretion, Absorption, Excretion, and Nutrition; completing, according to the author's plan, the description of the processes of organic life, "as far as those processes have for their object the conservation of the individual being." The subject will be continued in future volumes, by a description of the nervous and muscular systems, and an account of the action of air, water, heat, light, and electricity, on the different organized structures; and from this survey Dr. Smith purposes to deduce the proper rules "for the management of food, clothing, sleep, exercise (bodily and mental), and whatever other agent, under human control, influences the development of the physical and intellectual powers, and their due balance." When these subjects have been treated of, the consideration of the mental phenomena will be entered upon. This design, it will be observed, then, extends far beyond that of a mere popular book; being no less than that of influencing, through the earliest instructors, the minds of the young, with a view to increasing the amount of human happiness, by increasing their possessions in a department of unquestionably useful knowledge.

"Few," says the author, speaking of this extended view of his subject, "at present recognize its importance; but he who has a clear perception of the means by which human beings will advance to a higher and happier state, if they advance at all, can have no doubt that knowledge of this kind, made part and parcel of the mind, in consequence of having been received at a very early period of life, is destined hereafter to exercise an all-pervading influence over the speculations of the philosopher, the discipline devised by the practical educator, the institutions sanctioned by the moralist, and the laws positively commanded by the legislator." (*Pref.*)

The first chapter of this second volume (the eighth of the work,) is on Respiration. Dr. Smith sets out by stating that "no organized being can live without food, and no food can nourish without air;" and that, of the two, the necessity for the supply of air is the more urgent. He then gives a clear and pleasing account of the manner in which the air and the food of a plant react on each other in the leaf; explaining the digestive function performed by the leaf's upper surface, and the respiratory office of the lower. A fact often alluded to, perhaps without a very clear conception of its cause, is familiarly explained in the following passages.

"The physical agent by which this chemical change, which constitutes the digestive process of the plant, is affected, is the solar ray: hence the vesicles which contain the fluid to be decomposed are placed on the upper surface of the leaf, where their contents are fully exposed to the action of the sun; and hence also this process takes place only during the day, and most powerfully under the direct solar ray; but, although the direct influence of the sun be highly conducive to the process, yet it is not indispensable to it; for it goes on in daylight, although there be no sunshine. Light, then, would appear to be the physical agent which effects on the crude food of the plant a change analogous to that produced on the crude food of the animal by the juices of the stomach." (P. 7.)

The subsequent transmission of the sap thus prepared to the under surface of the leaf, where it is subjected to a process analogous to that of respiration in animals, is then described:

"This operation, which is strictly analogous to that of respiration in the animal, in which carbonic acid is always generated and expired, is carried on chiefly in the night. In this manner, under the influence of the solar light, the leaf decomposes carbonic acid; retains the carbon, and returns the greater part of the oxygen to the air, in a gaseous form. At night, in the absence of the solar ray, the leaf absorbs oxygen, combines this oxygen with the materials of the sap to produce carbonic acid, which, as soon as formed, is evolved into the surrounding air. The carbonic acid gas exhaled during the night is reabsorbed during the day, and oxygen is evolved; and this alternate action goes on without ceasing: whence the plant deteriorates the air by night, by the abstraction of its oxygen and the exhalation of carbonic acid; and purifies it by day, by the evolution of oxygen and the abstraction of carbonic acid." (P. 9.)

Dr. Southwood Smith's account of all these processes in the plant is written with his customary clearness and fulness of illustration, and leaves no part of them obscure on which physiology has yet thrown a steady light. He then explains to the reader the necessity of a greater extent and complexity in the respiratory apparatus of animals, arising out of their superior structure and functional activity; and goes on to describe the varieties of aquatic and aërial respiration; shewing that the simpler structure and limited range of functions of animals in the lowest part of the scale are associated with aquatic respiration, whilst the higher animals directly and more largely respire atmospheric air.

To whatever chapter we turn, we find the same minute and intelligible descriptions, and the same copious and valuable illustrations. In that on Digestion, there are numerous details concerning the process by which food is converted into nourishment in the various classes of animals, from the animalcules up to man. The descriptions of the human mouth, teeth, and instruments of mastication and deglutition generally, are admirably written; and the functions of the stomach and intestines are so perfectly explained that no reader whom we can suppose ever likely to open the book can fail to comprehend all that relates to the subject. At the same time all the higher and abstruser questions connected with all the subjects are so ably introduced that every professional student may refer to Dr. Smith's pages with benefit. This extent of real merit will yet, we fear, limit the popularity of Dr. Smith's volumes, which we should regret less on his account than on that of the public. His reputation will suffer nothing from being limited to very intellectual readers; but we should be glad to see the philosophical truths, interspersed with his anatomical and physiological details, spread abroad more widely; believing them to have an important relation to many social improvements which, in the absence of such knowledge, no benevolence and no patriotism can carry into complete effect.

II. With views more limited, with an ambition more easily satisfied, and with a distincter aim at certain classes of the " pensive public," Mr. Mayo, in a goodly drawing-room volume, inculcates the Philosophy of Living. He commences with an apology for inditing a popular book, an apology unnecessary in any case where the intention is to give useful views of subjects important to the populace; and especially unnecessary in Mr. Mayo's case, already distinguished as he is by his scientific writings. His object in this volume is to bring together and explain the rules which should be observed respecting Diet, Exercise, Sleep, Bathing, Clothing, and Choice of Residence; and to these he adds some observations upon Health of Mind; reserving for a separate volume, according to an odd custom of popular writers, some remarks on the Organs of Digestion. Mr. Mayo quotes, in his preface, the whole of Lord Bacon's Essay on the Regimen of Health, which we have ourselves always considered as comprehending more valuable knowledge than all the books on Diet and Regimen that ever were composed; but this wholesale quotation, of which there are several other instances in Mr. Mayo's book, lays him under the suspicion of book-making. Even book-making, however, may be venial, when the book that is made is good, and well adapted to a good purpose; and with a view to the part of the fashionable world who now and then look into well-bound publications, we pronounce Mr. Mayo's to be both sensible and useful.

Aware of the difficulty of defining the diversities of constitution, Mr. Mayo has yet thought it proper to devote some pages to this subject, under the heads of Temperament, Habit, and Diathesis; words, it must be confessed, often very vaguely employed. We cannot say that we think

Mr. Mayo has been much more successful in his attempt than those who preceded him; and we cannot but feel surprised to find him talking of such an imaginary entity as the *bilious* temperament. How odd it is that regular doctors will persist in reminding us of the witticism of the "Bath Guide:"

"I'm bilious I find, and my sisters are nervous."

What can be less definite than the information contained in the following paragraphs? (the *italics* are our own:)

"A gentleman with whom I am acquainted, *an eminent diplomatist*, is of the *nervous* temperament. He suffered some years ago with palpitation of the heart, which was *nervous*. A celebrated physician, lately deceased, attended him, and had him bled on two or three occasions; the treatment rendered him worse, and a different plan was adopted. His physician afterwards candidly advised him, whatever he did on the recurrence of these symptoms, not to be again bled.

"Another gentleman of my acquaintance, who displays the *nervous form of a mixed* temperament, with *excellent stamina*, a pulse slow and not excitable, about the middle period of life, when otherwise in health, became subject to nervous feelings, the most serious of which gave him the idea of sudden and imminent dissolution. He would occasionally wake in alarm with this impression, the feeling producing which was liable to recur during the day: Dr. Baillie told him not to mind these sensations, which resulted *from temperament;* and which, as he advanced in life, would leave him. They have done so." (P. 13.

The chapter on Diet is full of good rules, mixed up, it is true, with anecdotes from all imaginable sources. That on Exercise is equally distinguished by good sense and pleasant writing: it begins with a short and admirable explanation of the apparatus of motion, and to this succeeds much good advice concerning the exercise of the young and of the old. We are glad to find Mr. Mayo speaking very cautiously of the effects of what are called gymnastic exercises, in which the body is forced into actions much more suitable to monkeys than to man: "The exercises," he observes, "which pass under that name are inappropriate to boys; to the strongest, indeed, they do no harm, or may not; but children, not the strongest, are liable to be strained by them, and to have the seeds of permanent structural weakness sown by their influence." He speaks with approbation of "systems of exercises, in which the frame is thrown into a succession of attitudes, with gestures of more or less rapidity or force." Any quantity of exercise, he observes, may thus be obtained; "and there is this safety in such exercises, that we may be sure Nature has not overweighted the body; and this advantage, that, by means of an organized system of exercises, every class of muscles, every region of the trunk, each weaker or neglected part, may in its turn be submitted to strengthening discipline." (P. 120.) He does not fail to notice the defect of exercise, and its consequences, in girls: he considers girls as being healthier than boys, and liable only to one disease, "and that is Education." The following is Mr. Mayo's account of the spinal curvature so often induced in young ladies, of its causes and its progress.

"Mr. Shaw attributed the initial curve *to the loins;* Mr. Coulson is disposed to look for it *in the back and shoulder*. The latter writer, however, put forward, with a full general conception of its importance, the principle already quoted,—the general influence of the rightsidedness of our habits upon curvature of the back.

"But neither Mr. Shaw's nor Mr. Coulson's ideas are strictly just. *The two curvatures are not successive, but necessarily simultaneous*; neither has priority; in health and strength both of these curvatures, producing serpentine flexure of the spine, continually occur, and disappear with the next change of posture. In the weak, they become permanent, and are progressively aggravated.

"The steps by which the spine ordinarily gives way are these. The child kept at its music-stool, or books, or drawing, has a weakened and aching back. The muscles of the spine have not been invigorated by the sportive exertions and the various changes of attitude which nature dictates. Wearied by its task, the next change is to stand listlessly beside its governess, or in a drawing-room. What is the posture which it assumes? It is of course that which gives greatest ease to the languid muscles. The child stands with its weight supported upon one leg, the body swayed to that side, the knee of the other side bent, and the hip lowered. The limb which it uses on this occasion for support is almost always the *right* limb; for this simple reason, that it is the strongest. And the child assumes the position at all times, because it is one of change from its former more rigid position, and because, in addition, the fascial structure of the limb takes off, in that posture, some of the strain from the muscles. Let me, in passing, observe, that what has been already said sufficiently indicates the source of one minor kind of displacement, that is not unfrequent. The right ankle constantly rested on *grows* inwards,—that is to say, the joint *gives* inwards, its ligaments being elongated by the perpetual strain. In like manner, or from the same cause, the knee will give inwards,—one limb becoming in-kneed. The child thus weakened by its habitual inexertion, and tired by the discipline of the morning, is standing supported on its right leg. To judge of what is happening to its back at the same time, place before you a healthy child, and having instructed it to rest its weight alternately on both its legs, and, as in the position supposed, upon one only, observe its back when the alternation to the latter takes place. You may distinctly see that the straight line of its back becomes, in the second case, a serpentine flexure, *the ordinary flexure of curvature.* The mechanical elements of the change are equally obvious. At the time that the weight of the frame is transferred to the right limb, the left side of the pelvis is seen to sink; but the spinal column is attached at right angles to the middle of the pelvis; if the whole length of the column continued vertical to its base, the child would have fallen towards the unsupported side; the column, to avoid this consequence, is instinctively bent at the upper part of the loins to the right, to throw the weight well over to the right side; but the degree of flexion required for this purpose would carry the neck considerably out of the perpendicular; another contrary bend is therefore requisite, which begins in the middle of the back, and terminates at the root of the neck. These are the elements of the simultaneous changes which ensue, the inclination of the pelvis to the left, the flexure of the lower part of the spine to the right, of the upper part towards the left. They may be thus experimentally produced in the flexuous spine of the healthiest child, as quickly redressed, and the spine restored to straightness. *They are thus likewise inseparable, not successive, but simultaneous parts of one action.*

"Let us now apply the preceding observations to children with backs weakened in bone, sinew, muscle. This position of rest, this standing-at-ease, to which they are more prone than other children, and which becomes habitual, brings the spinal column into the following relation to the weight of the body, arms, and head. It is no longer a straight pillar of support; but, so long as the posture is maintained, a flexuous one. That would matter little if all the elements of the column were strong and rigid; but they are weak, debilitated, disposed to yield, and they give accordingly; and the flexures become, not the temporary yieldings of elastic joints, but permanent givings and yieldings of weakened textures. Once begun, the change can but progress, and the greater the obliquity at each part, the greater the mechanical inability of the spine to resist the growing evil. The only difficulty that remained in the theory of spinal curvatures was that which I have attempted to explain. When a curvature of the spine of the back is *convex* towards the right side, it necessarily follows that the right shoulder will be elevated, that the right side of the chest will be fuller, that the left shoulder will drop, the left breast be flat. It is easy, again, to understand how the case may be exactly reversed; how the habitual inclination may be to rest on the left leg, with a parallel train of consequences. Again, it is evident that the serpentine curvature, the *common* origin of which I have explained, may be favoured by or even entirely proceed from other causes; how an invalid in bed may be bent with a convexity towards the side on which she lies; how the spine may be twisted slightly on its axis by one or other habitual motion; how the posture employed in writing or drawing may give rest and pressure to one side and shoulder, expansion and elevation to the other; how the trivial circumstance of the dress of children hanging on their shoulders, and so contrived as to be always ready to fall off, will almost necessitate the practice of perpetually hitching up one shoulder to support the dress, and of letting the other drop within it. The description of the steps by which weakness of the back leads to curvature, explains at the same time the means of preventing the latter, or of remedying its early threatenings." (P. 126.)

In connexion with this most important subject, we request the reader's attention to Professor Stromeyer's views given in our last Number; p. 133.

Some observations occur in the chapter on Sleep which, as their practical bearing is extremely important, should be received, we think, with more caution than they are given. Speaking of the condition of the brain during sleep, Mr. Mayo quotes a case from Blumenbach of a person who had been trepanned, and whose brain was observed to sink when he was asleep and swell out when he was awake; and this is considered to be a proof that when we sleep the cerebral circulation is more languid than when we are awake. Mr. Mayo says that it is no less certain, although it is difficult to prove, that "the nervous power of the brain is lowered in sleep." The bearing of these conclusions is, he observes, of supreme importance. "Brain attacks" commonly occur in the night; and, consequently, at a time when the power of the brain is lowered. To bleed, therefore, in epilepsy, apoplexy, and palsy, especially in persons advanced in life, is not the proper treatment; these attacks result from general or partial cerebral failure: they require repose of mind, gentle exercise, free air, a regulated diet, sometimes medicine, sometimes stimulants. They may, however, Mr. Mayo acknowledges, be primarily induced by "*action in the head*," and may often be traced to full living and excitement, and sometimes the circulation is loaded and labours, and in these latter cases, the diminution of the quantity of blood by cupping is most likely to give relief. There can be no doubt that in aged persons attacks apparently apoplectic occur from causes not requiring or justifying depletion; and that in many cases of epilepsy and palsy, and in the convulsions of children, bleeding is positively injurious, and an opposite treatment beneficial. Any conclusions drawn from the occurrence of such seizures during sleep, however, would, we fear, prove too unsure to be relied upon.

In the chapter on Climate, there is an interesting section on the climate of the factories. Mr. Mayo quotes Dr. Ure's account of the state of the work-people, by which it would appear that under the influence of the steam-engine, the blest genius of the place, crowds of feeble persons are enabled to earn a livelihood, screened alike from summer's heat and winter's cold, and thus procuring abundant food, good clothing, and what Dr. Ure is pleased to call "domestic accommodation." These views, as Mr. Mayo justly remarks, rest on partial facts, and do not comprehend the general condition of the manufacturing population. He cites what we conceive to be the juster opinion of the late Mr. Thackrah of Leeds, who observed the children to be "universally ill-looking, small, sickly, barefoot, and ill-clad." The young men were pallid and thin as children: none of the women fresh-looking; altogether, "a degenerated race—

human beings stunted, enfeebled, and depraved." This evidence is strikingly confirmed by the recent testimony of Mr. Wing, part of which we shall here quote :—

"Having provided myself with a thermometer," says Mr. Wing, "I visited ——, a coarse mill, and found the operatives, both adults and children of thirteen, worked the twelve hours, exclusive of meal-times,—that is to say, from six o'clock in the morning to half-past seven at night, with half an hour for breakfast at eight, and one hour for dinner at twelve; about four another meal is taken, without stopping the machine. A copper of hot water on the outside supplied the power of having tea and coffee, which the operatives thus obtain: the different parties, as they come, unfold a paper containing tea or coffee, with a little sugar; the contents are placed in their pitchers, the water is poured upon the ingredients, they quickly depart to their different operations, and can scarcely be said to rest at this period of refreshment. In the card-room, a boy, who was in-kneed, sickly-looking, short, and of spare muscular development, and apparently about twelve, I found, to my astonishment, was seventeen years old. 'Had worked five or six years in a mill. On a Monday morning was so stiff could hardly move, and at night was sadly tired; his limbs were straight when he entered the mill.' This unfortunate boy was doomed to work the full time. The temperature of this room was sixty-seven. A boy belonging to this mill having told me he was thirteen, upon being closely interrogated, said that he should be of that age the next month; he worked the full time: my belief is, he was barely twelve. In the dressing-room of this mill there were nine adults, who looked of a factory cast. The temperature was ninety-eight degrees, and the overseer said that when the gas was up, the increase of heat would be to 103 or to 105 degrees. The work necessarily required a high temperature." . . . "In one mill which I visited, there were about forty operatives in the card-room, which was very dusty, and oppressive to respiration. I found the effluvia particularly offensive. To describe the effluvia in some of the rooms is difficult, partaking, as it does, of the combined qualities of the friction of the machinery and of oil; the effect is of a faint and sickly nature. I had an opportunity of seeing spinal deformity, and, upon making the relative enquiry, I found that it was induced by the system, and I saw wounds caused by the machinery." (P. 265.)

It is evident, from these and other passages which might be quoted from this work, that, notwithstanding the increase of popular knowledge respecting health and disease, the principles of justice, a compliance with which would secure larger portions of the community from the penalties of bad air, bad food, and all the evils of poverty, are not yet generally complied with or even commonly adverted to. It is not our duty to dwell, as Mr. Mayo has very properly done, on the moral evils incidental to those who live and labour in the factories. We trust that some among his fair and fashionable readers may be led to reflect on the lot of women especially, who go to their toil from home at very early hours, return only to hurried meals, see little or nothing of their children, preserve no domestic habits, and bring up a young family amidst the disadvantages of an enfeebled frame and almost continual absence from the little creatures who demand a mother's care. Perhaps, too, although on this point we are more doubtful, their sympathies may be roused for the poor infants consigned to mercenary nurses, and early taught to drink laudanum and gin. An occasional word of compassion, addressed to legislators by those who seldom speak in vain, would do no discredit to the female character, and be in no degree opposed to the principles of Christianity. But how seldom are such words spoken! In this portion, and in many portions of his book, Mr. Mayo's sentiments do him great honour; and we feel both respect and gratitude for his endeavours to awaken good feelings as well as to establish good habits among the readers of his elegant and sensible volume.

III. Mr. Mayo's supplementary volume, entitled "Management of the Organs of Digestion," is remarkably illustrative of the little necessity that existed for divorcing it from the former. Seventy pages of it are devoted to diet and indigestion, including several subjects which shew the difficulty of filling even these with appropriate matter, such as sickness of pregnancy, sea-sickness, and vomiting following surgical operations. About forty pages more are devoted to the management of the bowels; and here the book should have ended; or rather, all this, or part of it condensed, should have been added to a condensation of the former volume. The consequence of the contrary plan has been, that when Mr. Mayo arrived at the 109th page of this promised book he found he had nothing more to say on the proper subject of it, and was compelled, against his better judgment we doubt not, to treat the public with an account of all sorts of diseases of the rectum. Of the value of this part of the work we do not speak, but at the best it can only be considered an addition to a popular treatise—made in very indifferent taste.

Mr. Mayo gives some interesting cases in which symptoms, occasionally perhaps mistaken by practitioners for indications of fulness of blood, arose from want of proper nourishment, including one in which epileptic fits were apparently kept off by timely stimulants. Other examples, less liable to be mistaken, are given, in which chilliness on going to bed and a thready pulse, pointed out the propriety of taking some moderate stimulant at night; and it is remarked, judiciously we think, that as those who have mental exertion to go through after dinner, are indisposed to do so by a hearty meal, and yet not sufficiently supported by a very light one, a light supper may be indulged in by them without any impropriety. Some examples are also given of the bad effects of too rigid a diet, with abstinence from animal food, and also of too vigorous antiphlogistic measures, in inducing not only fainting fits, but fits of epilepsy. These are cases to which the young physician will sometimes find himself called in by his elders among the general practitioners, and great firmness is required, as well as discrimination, to prevent a perseverance in the bold measures which have done all the mischief. Vague notions of congestion, or of what is still more vaguely called "general inflammation," beset the mind of the lover of the lancet; and as the symptoms are generally serious, and the result of a wrong decision may be death, the physician has need of all his observation. We have known symptoms relieved in half an hour by a moderate stimulant, for which it had been strongly contended that a large bleeding was essential. The colour of the face, the state of the pulse, and all the circumstances indicative of excitement or exhaustion must be carefully observed and compared in these cases; and the theories of the unreading men, who have their theories no less than the scholar, put aside. The number and variety of symptoms arising sympathetically, and depending on disorder of the stomach, must never be forgotten. Cases resembling paralysis, and even apoplexy, are sometimes of this kind: but then the observer is not to set down every case as sympathetic of stomach-disease. No disquisition on these subjects in a popular work, we may observe, is very likely to be auxiliary to the practitioner, who may vainly contend with the prejudices of the lady of the house, or a clergyman "who knows something of medicine," and has even read Mr. Mayo's book. The following case, degraded to a foot-note, was worthy of a place in the text. It is illustrative of the effects of a cause merely mechanical.

"Dr. Sweatman attended a lady who had been compelled to remain in the horizontal posture for ten years. If she sat up, in a short time she fell into, or began to fall into deliquium, and fainted; but she looked in perfect health, and had borne several children during this period. The opinions of several eminent London physicians had been taken, who, finding nothing wrong in the circulation, had all recommended tonics. Dr. Sweatman begged to see the patient's legs, which he found covered with immense varicose veins. Into these, when she stood up, all the blood in her body gravitated; and hence she became faint. The legs being bandaged, she was cured at once." (P. 15.)

We cannot imagine a case more forcibly illustrating the necessity of a careful examination before prescribing; or one more calculated to put all those who had been previously consulted very much out of countenance.

There is much sound sense, we think, in Mr. Mayo's remarks on the effects of a lowering plan of diet and antiphlogistic treatment too long pursued after surgical operations. The ill consequences are spoken of as more common in the French hospitals than in our own; which we can readily believe, having witnessed, we fear, the absolute starving to death of some men, some women, and many children, in the Parisian hospitals; but the fault and its consequences are not unknown at home. We see, or used to see, patients sinking after operations from what the older surgeons called "an indescribable state of irritation," which supervened; and we have witnessed even livelier examples in the country. A sportsman, in the prime of life, and the vigour of health, hunting four days a week, and taking a bottle of claret after an excellent dinner daily, and not living very poorly at breakfast, has a fall with the hounds, and breaks his collar-bone, or dislocates his shoulder. Pale and faint, he is hurried home, and surrounded by surgeons; the shoulder after some trouble is replaced, or the collar-bone attended to. Comfort and warmth return: blood is taken; calomel is given, a black draught follows; gruel and tea are allowed, but nothing better; no dinner, no wine. All this is well, or even quite necessary; but in a few days, more or less, it is neither well nor necessary. The patient begins to feel the exhaustion produced by his sudden low diet, and his doses, repeated too often, of calomel and draughts. The dinner comes, no dinner hour to him; no wine revives, no evening company consoles him. Both mind and body suffer. He becomes irritable, restless, yawning, very sensible to noises, sleeps ill, and thinks the day as well as the night will never end. He becomes alarmed, and sends for his surgeon. Even in these cases bleeding is sometimes proposed; and we have seen it proposed to an extent which we think would have been fatal, and in which, at least, the stimulus of strong coffee, or a moderate dinner, and a little wine, would have entirely restored the patient to feelings of comfort.

Critics more captious than ourselves might take exception at the manner in which the information contained in Mr. Mayo's book is frequently brought before the reader. "A very learned and distinguished person told me." "An artist of eminence I had occasion to prescribe for." "A gentleman now in his eightieth year, who is under my care for a local complaint." "A few nights ago a gentleman sent for me." "In a case, in which I performed amputation at the hip-joint (the patient happily did well,) for a few hours the utmost prostration existed," &c. &c.

These we look upon as inadvertencies, which to be disapproved of need but to be seen; but seen too oft, they are endured, considered allowable, and imitated. The gentleman who sent for Mr. Mayo " a few nights ago" had a violent fit of ordinary indigestion early in the morning, and was cured by an emetic of salt and water, of which Mr. Mayo speaks very favorably. Half a teaspoonful of common salt is given in a tumbler of tepid water, and a second in a few minutes. This is said not only to cause vomiting of the offending matter, but, if it has already been in a great measure got rid of, to calm the stomach, and to act as an aperient. These slight attacks of indigestion, it is observed, if neglected, are succeeded by diarrhœa, or by an indisposition of several days, with loss of appetite, heartburn, a furred tongue, and general uneasiness. Mr. Mayo gives ample and judicious directions for the treatment of what are generally called, with doubtful propriety, bilious attacks: but it might have been well to warn his readers, and particularly his London readers, and more particularly still, his lady readers, against the habitual resorting to calomel and black draughts; which, producing immediate relief, are often had recourse too so frequently as to convert an occasional into an habitual invalid. The use of a simple aperient pill, followed by a domestic enema, or even an occasional enema without any pill, is well adapted to cases in which torpor of the bowels characterizes the intervals between one of these favorite doses and the next; and will generally render calomel and strong medicine unnecessary, to the great comfort of the intestinal canal.

The plan of Mr. Mayo's book seems to be to teach by the introduction of cases; which are given to exemplify the state of habitual indigestion, and the effects of particular kinds of diet in relieving very aggravated suffering, although no general rules can be deduced from the relief afforded in particular instances. He thinks it superfluous to enlarge on the good effects of bodily exercise, friction, change of occupation, mental relaxation, and amusement, with change of air and scene, as means of cure; but makes more particular observations on the use of aperients, including the dinner pill, that ingenious invention of luxury, unwilling to forego enjoyment yet anxious to escape the penalty of excess. He recommends it in the shape of four grains of rhubarb and one of carbonate of soda; and a night pill of equal parts of Castile soap and compound extract of colocynth; " or the same," he says, although we do not know whether he means the dinner or night pill, with two grains of scammony, or, instead, a drachm of the lenitive electuary; " or, in the morning, a drachm of Epsom salts in peppermint water, alone, or with two drachms of tincture of rhubarb; or two drachms of Cheltenham salts, in a third of a pint of water." (P. 43.) These directions remind us of an acute saying of Mr. Landor, that, in conversation, people do not expect to be instructed, but to have their own ideas reflected. We know many habitual dyspeptics who take all these things, dinner-pill, night-pill, morning draught, and occasional electuary , but few who are the better for them. The public require to be told that medicines of this kind are but the substitutes for the common sense which would dictate exercise and good regimen; and that when the auxiliaries of rhubarb, and soap, and Epsom salts become habitually necessary, the case of the dyspeptic is almost hopeless. The purgative school has, we are convinced, much to answer for: every day we meet with its victims even among the young, particularly among young ladies, who are sent to school with a box of family pills, just as regularly as with a spoon, silver fork, and the other requisites. To induce them to abandon the practice of pill-taking, and to enable them to do so by the substitution of exercise before breakfast, and at other intervals during the day, is to save many of them from being invalids all their lives, and almost useless to their families, if not plagues to every body about them.

It is well observed by Mr. Mayo, that a combination of tonic remedies with purgatives is often advisable; and he quotes a striking case of gastrodynia, from Dr. Abercrombie, in which nothing proved, after the trial of many medicines, of so much use as a combination of two grains of sulphate of iron, with four grains of aromatic powder, and one grain of aloes, taken three times a day. These are the cases, so often met with by the physician, in which the unfortunate patients have taken all kinds of mercurial preparations, not only without advantage, but with infinite detriment.

Mr. Mayo considers the disorders of the stomach with reference to its secretion, its mode of sensibility, and its action. Female readers, afflicted with spasms of the stomach arising from any of the little contrarieties of life, will be glad to find themselves advised to have recourse to sal volatile, brandy, until "wind breaks off the stomach," and, if these fail, to take a few drops of laudanum. There can be no question that all patients of this kind will entertain a very high respect for Mr. Mayo's opinions. He warns them, however, against indulging in increasing doses of the latter remedy; and does not forget to observe, that pain of the stomach, although often a trivial malady, may depend on serious disease. But, still inclined to make the readers their own physicians, he tells them, if there is tenderness on pressure, which he admits may indicate ulceration of the lining membrane of the stomach, to apply leeches and blisters, and to take the milder preparations of mercury; using, at the same time, abstinence. We have nothing to object as regards the remedies; but what can be said of placing them in popular hands, when fatal disease may be impending? So also, with respect to the subject of vomiting, the directions are generally good, but it can be of small use to the popular reader to peruse cases in which this symptom yielded to "sugar of lead and opium."

The observations on the different forms of diarrhœa, and their management, are plain and practical: but we are surprised to find Mr. Mayo asserting (p. 86) that "the indigenous cholera of this country is characterized by purging of bile;" and the account of Asiatic cholera is surely somewhat uncalled for.

Among the effects of habitual constipation, Mr. Mayo enumerates indigestion in all its forms,—every disease of the rectum, headach, hypochondriasis, fulness of the cerebral vessels, palsy, and apoplexy. Mr. Mayo is a greater advocate of the habitual use of purgatives than we profess to be. The importance, on "many occasions," of using "the principal means that are calculated to correct this complaint (constipation) when it does not exist;" and the "value" of aperient medicine "in numerous cases where the bowels are not costive," are, if truths, such as we are scarcely prepared to receive. There are, no doubt, some cases that might be cited in support of such a doctrine; as that of an existing tendency to plethora; but we fear that this opinion, if even medically true, is not devoid of danger applied to popular practice; whilst the rule which Mr. Mayo deduces from the supposed principle, that "when the system is out of order, and there is no failure of stamina, in three cases out of four it is only necessary to purge; that is to say, to excite great increase of action in this one secretory organ,—and the disorders of the rest subside," (p. 100,) is, we are perfectly convinced, pernicious in a high degree. The theory, often as it is acted upon, is seldom so openly avowed; and the avowal, in the work of so distinguished a physiologist, will be very welcome to many a student and many an idle practitioner; leading to a compendious and ready practice, without the fatigue of thought or the anxiety of doubt: but we question the innocuousness of the practice, nevertheless. It may often relieve present troubles, but it lays the foundation of others, which will rise up in after years; and, as we suspect, among them some of those general and local affections of the bowels which occupy so considerable a portion of Mr. Mayo's volume. Indeed, for the relief of occasional constipation, Mr. Mayo does not himself advise medicinal interference. Where it arises from neglect of bodily exercise, the cure, he says, is easily suggested; and walking or riding before breakfast is most salutary. Thus far we agree with him; but not when he recommends afternoon exercise as preferable for women, and approves of seven o'clock as a dinner hour, because it allows "from ten to five for business, and leisure for exercise after it." (P. 101.) Such recommendations are very agreeable to fine ladies, and to their busy, excited, ambitious husbands; but they are not, we conceive, quite so useful as agreeable.

The following observations are of much practical interest, in relation to mechanical means now very frequently resorted to in this country, and long almost universal in some parts of the continent.

"Those in whom fæces are formed in adequate quantity, but are not voided through sluggishness of the muscular structure of the bowel, or because there is not liquid enough, often obtain perfect relief by the use of injections of water. The water should be used cold, to be more stimulating; to render it still more so, half a teaspoonful of salt may be added. The quantity ordinarily requisite is a pint. It is sometimes, of course, proper to use warm water. Many persons, who are temperate livers, and of sedentary habits, find the daily use of such an injection, half an hour before the customary time of relieving the bowels, sufficient to obviate troublesome costiveness, and to cure the local complaints it has produced. But this remedy does not answer with all: in some it produces uneasiness and a sense of weight and dragging from the loins, with general lassitude; in others, the bowel becomes relaxed, and a disposition to protrusion is formed through it. Nor is the practice of injections wholly free from more serious accidents. I have known the intestine torn, and the gravest consequences ensue, through inexpertness in the use of the instrument. The tube of the apparatus is generally made so long and sharp, that it is easily capable of tearing and lacerating: properly, it should not exceed an inch and a half in length, and should end in a smooth sphere, half an inch in diameter, tapering to a neck a third of an inch thick." (P. 102.)

The remarks made on the administration of different purgatives require no comment from us, except that we fancy we discern the same accommodating conformity to popular notions which we have already taken the liberty of noticing, when Mr. Mayo says there are some persons whom "mercury always suits;" and that, with these happily constituted individuals, "it serves as the best alterative, the best aperient, and, indirectly, the best tonic;" all which must be considered very questionable.

The general subject of the present article excludes us from any particular notice of the remainder of Mr. Mayo's book, which constitutes nearly one-half of it, and comprises Fulness of the Hemorrhoidal Vessels and Bleeding, Piles, Prolapsus, Fissure of the Rectum, Contraction of the Anus, Stricture of the Rectum, Substances in the Rectum obstructing the Passage, Defects of the Lower Bowel existing at Birth, Laceration of the Intestine, Abscess and Fistula, and Cancer of the Rectum. On all these matters, whatever may be thought of laying them before general readers, we do not doubt that every reader will find very useful practical remarks in Mr. Mayo's pages; and his general observations on the causes and prevention of affections so peculiarly distressing to many who would otherwise employ the later years of life in pursuits the most worthy of rational beings, but are condemned to daily suffering and wretchedness in consequence of some of them, are valuable to all readers. He lays the greatest stress on abstemiousness; on avoiding the habit of costiveness, and on a scrupulous observance of cleanliness. All the rest, we think, would have been better omitted; for, if these means fail to prevent the occurrence of the lightest among the disorders of the lower bowels, the only security, as regards continued comfort, is to be sought in the advice of a physician or surgeon. As some of these disorders are also often imagined to exist by the nervous, we fear Mr. Mayo's book will do some harm to a few of his readers, unless it leads them to have recourse to professional opinion.

IV. In our notice of the two volumes from which we have just parted, we have entered so fully into the means of preserving and restoring health recommended by their author, that we may be excused for not taking up the subject again on turning to the work of Dr. Johnson, which is consecrated to the same benevolent objects: and we the more willingly adopt this course, because what we shall have to say of the *manner* of Dr. Johnson's volume will leave us but little space to animadvert at any length on its matter; which, however, must be allowed to be, on the whole, both entertaining and valuable. It is not very often that we dwell minutely on the mere style of an author. The advancement of medical knowledge is so great a benefit to the public that it may well excuse many literary defects in any writer; although, generally speaking, we ought to expect that a medical author should express himself like a man of education. Every man has his own style, formed by his course of reading and his modes of thinking; and his own style is best suited to his own thoughts. Even in the intricate sentences of John Hunter, we seem to perceive a great genius making his way through the fogs and mists which hang about other men's minds. But we consider that we have a right to look for a correct manner of writing and a style befitting a learned profession, when a physician takes upon himself to speak for his brethren to the general public, and to explain to them what rules of health the studies and experience of his colleagues have succeeded in establishing. We do not like the excellent wisdom of medicine to be disfigured with gaudy ornaments, and truths of great importance to the happiness of society to be delivered in the slang of drawing-rooms, or the gossiping dialect of nurses, or the wretched drivel of feeble-minded and pampered hypochondriacs. Medical science is then degraded from its true rank.

The truthfulness of criticism compels us to say that we have fallen into this train of reflection on looking over the pages of Dr. James Johnson's "Economy of Health, or the Stream of Human Life from the Cradle to the Grave." He disappoints our reasonable expectations. The general aspect, the very title-page of his book, has that suspicious, or, we should say, that meretricious character which a man, anxious for the true respectability of his profession, cannot remark without regret. We recognize the great model, of which, in the course of our critical duty, not a few imitations have come before us. The imitation of such things is indeed easy, requiring only a negation or absence of all estimable qualities of manner: but how such a model can be exhibited by a physician possessing so many excellent qualities, largely experienced, not to be suspected of a want of information, and accustomed to the daily collision of a world in which good sense is not quite trampled under foot, we are really at a loss to explain. Not in this work alone, but in Essays on Morbid Sensibility, and on Change of Air and the Pursuit of Health, and on Autumnal Relaxation, &c. &c. we discern, too plainly to be denied, the same catching at the folly and weakness of the day, and the same appeal to those whose approbation confers small honour. The results are curious: in each we turn away, wearied, and more than wearied, by such a vertiginous commixture of subjects as no brain can bear. In every one, and in almost every page, we see the same violent quotations of the poets,—the same unnatural intertwining of Latin and English,—the same literary impromptus made at leisure,—the same humorous headings of chapters,—the same dashes, hyphens, notes of admiration, and other wonders of the printer's art,—the same lavishness of capital letters,—the same comprehensive type, and the same somewhat indifferent paper. But the strangest thing of all is, that we find much evidence of strong good sense, a tone of experience not to be mistaken, and, assuredly, a bonhomie which disarms the severity of criticism.

There are of writers not a few, to whose reputation it would do great service if they were occasionally debarred for a time from pen, ink, and paper. It is undeniable that, with the author whose peculiarities now occupy our attention, writing has become a habit; and there are probably few intervals of his busy life in which he does not write out every thought that passes through his active brain. By a fatal facility, every thought thus written finds hasty way to the printing-office, and is fixed in types for ever. As must happen, and always does happen in such cases, the first works are the best; for authors of this kind always contrive to let themselves gently down, by beautiful degrees, until they perish wholly by their own recording hand. There is much that is worthless in their first compositions,—more that is worthless in the second,—and the adjective is subjected to its superlative degree of comparison in the closing portion of the series. The subjects of the later works, being anxiously sought for, necessarily become more forced; the quotations grow more distressing, the dashes are more desperate, the headings of chapters more wilful and barefaced, and the capital letters more outrageous. Even the good sense can scarcely hold out for ever, or, as Dr. James Johnson would say, "to the crack of doom;" and the bonhomie gives place to a bristling, challenging air, defying critics who delight to find fault with original writers. Such was once the career, and such the fate, of Dr. Kitchener, as may yet be instructively seen in his Art of Invigorating Health, his Peptic Precepts, his Principles of Cookery, his Treatise on Spectacles, and on Reading and Accent, and a score of other works which had their glittering day. Such, and so oblivious, will be the fate of Dr. Johnson, unless his good sense comes to the rescue. Without that help, his literary life will never, to quote one of his felicitous creations, reach its Tenth Septenniad.

The Economy of Health presents more than its share of the faults we have tenderly alluded to. In a few pages of introduction, staring capitals meet the eye, blazing forth the words Health—Time—Poverty—Affluence—Fame—Power—Sceptre—Prince—Beauty—Literature—Science—Religion—Cholera—Hygiène—Community. These words, starting out from undistinguished type, are well-adapted appeals to idle people, who open new books in publishers' shops; and no critic, however lenient, can fail to perceive that, being interpreted, their signification is, Try Warren. Then, health being happiness, Pope of course is quoted,—"Oh happiness!" *et cetera;* and, fame being alluded to, we have "the pillow of Napoleon." The Hindoos, the Egyptians, the Hebrews, the Greeks, and the Jews, are not forgotten. Lord Byron, Ajax, Ulysses, Lycurgus, Xenophon, Pythagoras, and Sir John Sinclair, shine together; and the eleven pages into which this galaxy is crowded end with the particularly unnecessary and novel assurance, in any work of the author's, that he shall embrace

"Quicquid agunt homines," &c. &c. &c.

It must be, that, after Dr. Johnson has written the *sense* part of his books, he entrusts his pages to some engrafter of poetry. No sooner is Pope done with, than unhappy Goldsmith is seized upon: "The soul's dark cottage," and so forth; then comes Horace; then Goldsmith again; then Horace again; then a French monarch; the old story of "*toujours perdrix*," in capitals too; then Shakspeare; all in twenty more pages. This is the very wantonness of literature.

Turn where we will, the same sportiveness confronts us. Is the fashionable world mentioned? straight we have "the gay, licentious proud." If love is spoken of as a disturber of life, he comes as Cupid, with Nox and Erebus, with the Upas, and with Juvenal and Persius; and then we have Shakspeare, with "She never told her love;" and, worse still, with this abominable addition, "*but* Shakspeare knew not a tithe of the numerous links in that extensive chain," &c. &c.; which shews how much more easy it is to quote Shakspeare from Dodd's "Beauties" than to read, mark, learn, and inwardly digest him. Who but Dr. Johnson's assistant quoter could have said of Shakspeare, that "he knew not a tithe"—of anything?

Are young men exhorted to be prudent as to marriage, it is "Parce puer stimulis et fortius utere loris." Do we speak of drink, we have Circe and the turning of the friends of Ulysses into swine. Is the choice of a wife alluded to, we have "the lunatic, the lover, and the poet," and two quotations more. Is ambition touched upon, we have "Cromwell,

I charge thee fling away ambition." Does Dr. Johnson faintly surmise that it has pleased God to give a tolerable brain to rustics, as well as to ladies and gentlemen, we have two stanzas from Gray: " perhaps in this neglected spot;" and, at p. 176, we are a second time assured that the work embraces " Quicquid agunt homines, votum, timor, ira, voluptas;" which would appear from this to be a very favorite quotation. Speaking of the mingled pain and pleasure of revisiting scenes known in early years, when we have reached the "seventh Septenniad," it is recommended that we confine our sympathies with such spots to letter-writing; and we should feel surprise here if Dr. J. did not tell us that "Heaven first taught letters:" but, turn over the leaf, and there we have the very quotation, and with the illustration of capitals: " Heaven first taught LETTERS," as if the poet meant the documents concerned in the projected Post-office reform. Speaking of memory, he passes, by a rapid transition, to the havoc which BEAUTY (in capitals, of course,) makes in the memory of other qualities, and then we have

" If to her lot some female errors fall," &c. &c.

Half a page further on we have four lines more from Pope, most of whose works will be found quoted from beginning to end, we imagine, in some or other of Dr. J.'s volumes.

The number and nature of these quotations is not excused by the manner of their introduction. Few of them arise naturally out of the subject. They do not so much appear to be the recollections of a mind filled with classical images, as to be dragged in perforce with " it is said by the poet,"—" our poet has remarked,"—" the words of the poet," &c.; a phraseology as plainly indicative of the effort to enrich common matters with extrinsic embroidery as can well be conceived. The broken lines amaze the ignorant and vulgar, but give no satisfaction to readers whose approbation is far more to be desired.

But enough of this. Perhaps we take up the subject of these blemishes too seriously: they may only appear so to us. They may be caused by Dr. Johnson's more correct estimate of the mass of the reading public; and of the hypochondriacs, valetudinarians, and wretched sensualists who walk the Steyne or the beach, the pump-room or the promenade, in search of that happiness which they hope to build without any foundations, or who knock at the doors of fashionable doctors to seek deliverance from their ill-regulated minds and pampered bodies. But to such, and such alone, Dr. Johnson's pages seem adapted; and we hope that these unhappy readers, at least, may be able to gather the real sense so ignominiously overlaid. We are tempted to be ourselves figurative, in the author's line. The fine lady congratulates herself that she takes pills unknown to vulgar palates; not smelling of base orris-root, or sticky with starch, but enveloped in leaf of gold. The faithful aloes and stedfast rhubarb, nevertheless, perform their duty, and, as Dr. J. would remark, "cleanse the full," &c. &c. &c., as " our poet says," without respect to rank or station. Dr. Johnson's style is the gold-leaf, glittering but valueless; and, if his practical experience acts a salutary part, despite the tinsel, on the sickly minds of Tonbridge, Hastings, Cheltenham, and Leamington, well and good. Upon such minds the sober reflection, the profound acquirement, the dignified simplicity, which we have been accustomed to regard as the proper characteristics of a physician's writings, would be quite thrown away.

With these drawbacks, which some may consider trifling, Dr. Johnson has composed an amusing, desultory book, upon every possible subject. Ten chapters embrace nine septenniads, the fifth and sixth being comprehended in one, and each septenniad has its characteristic objects for remark. The fifth and sixth septenniads form one chapter, and this chapter is headed The Golden Æra. Out of forty-two subjects, distinctly referred to in this chapter alone, we select, from the table of contents, a few, in order to shew the discursiveness of the author's manner. This extract gives also a few illustrations of the compound terms and types.

"Development of a grand Principle in Hygiène.—Activity of Body as an Antidote to Depression of Mind. Illustrations.—Retreat of the Ten Thousand Greeks, under Xenophon.—Siege of Mantua.—Shipwreck of Captain Byron.—Retreat of Sir John Moore.—Narratives of Bligh and Wilson.—Retreat of the French from Moscow.—Application of this Principle of Hygiène to Private Life.—Græco-Byronian Precept, 'Keep the Body active, and the Stomach empty.'—Misfortunes of the Female Sex.—Ingratitude to Mothers.—Maternal Affection.—Filial Affection.—Punishments in this World.—Suicide.—Hope of Rewards.—Zenith of the Journey of Life.—Retrospection.—Tree of Knowledge.—Probable Effects of Knowledge.—On Intellect.—On Learning.—On Wealth.—On Rank.—On Happiness.—On Equalization."

V. Dr. Robertson, of Chesterfield, has compiled a sensible and useful book on Diet, Bodily Exercise, Ventilation, Climate and Change of Air, Dress, the External and Internal Effects of Water, Mineral Waters, and Sleep. This may probably be regarded as one of the numerous works for which the world is indebted to Dr. Combe's admirable publication; but Dr. Robertson has contrived to give a novel air to this subject by his arrangement, and by variety of topics. It is written in a plain unaffected manner, and contains much valuable information. The appended tables of mineral waters give it additional utility to many readers; and Dr. Robertson's observations on the subject of these highly fashionable remedies are instructive and judicious. Seeing so much as we do of the capricious manner in which mineral waters are recommended and taken, we value every fragment of evidence concerning any of them which seems really authentic, as the following, relative to Buxton, appears to be.

"It was my fortune to see Buxton, for the first time, after riding twenty-four miles, the greater part of the journey having been performed in the heat of a summer's day. I dismounted, and in less than five minutes was in the act of undressing for a plunge. The baths are fitted up with great neatness, and are lined with white porcelain. Water is constantly running through them; no small advantage to the man of nice and refined ideas. Perhaps the best thing that I can do is to state briefly the effect which the water had upon myself. On plunging into it, the shock was unusually severe, greater than I ever experienced in ordinary cold bathing; of course greater than I ever felt from bathing in the sea: and this is not a thing peculiar to myself, but, I believe, the usual effect that these waters produce. Reaction, however, came on much quicker than is usual with me in cold bathing; and this was followed, in about ten minutes, by all the luxurious languor which the warm bath produces. This increased until I left the water, (in twenty minutes,) when, expecting to find my skin easily chilled, as it usually is after warm bathing, I was agreeably surprised to find it all in a glow, my strength recruited, my spirits even buoyant, and my appetite voracious. I trust this little bit of egotism will be pardoned; for really so few, except those who go to a watering-place, know anything of its effects, that it may be useful." (P. 191.)

Dr. Robertson omits to mention the temperature of the water, which is stated in the table at the end to be 82°. He considers the baths of Buxton favorable to all whose cases require a course of temperate bathing, combined with the advantage of a somewhat bleak but bracing air. We very much approve of his observations on the insufficient knowledge yet possessed of the actual qualities of mineral waters, or of the mode in which the ingredients exist in them, by mere chemical analysis. The common idea is, that the water which produces, by analysis, the greatest quantity of sulphate of magnesia or sulphate of soda must be the best, because the most purgative; and patients resorting to Cheltenham and Leamington cannot be persuaded that any other benefit is to be expected than that of acting briskly on the bowels. The impatient desire of the sick for immediate results presents a great obstacle in the way of investigating the effects of various doses of many medicinal substances. Without being followers of Hahnemann, we know that several drugs are given in doses much larger than necessary, and that smaller quantities are more serviceable. Many a man is deterred from giving small doses by the fear of being esteemed a timid practitioner; but, if a few grains of medicine cure a disordered condition of body, we see no glory in the administration of the same medicine in ounces and pounds.

It is indeed strange, as Dr. Robertson remarks, that so much of the real benefit of watering places being produced by the altered habits, improved regimen, and greater activity and cleanliness attendant on the change, it is yet so impossible a thing to induce the same dietetical and regiminal reforms at home, without trouble and without sacrifices; but it is still to be remarked, that the mind is refreshed by change of place, relieved from habitual cares, and rendered capable of more exertion than it can put forth at home, whatever the doctor may advise, or whatever the growing symptoms of debility may threaten. There is no man, whatever his resources or his strength of mind, who does not derive some benefit from change of place; and the facilities now created of obtaining this kind of change will probably be very beneficial, among other things, to the public health.

VI. Dr. Paris's Treatise on Diet is already too well known, both to the profession and to the public, to require any lengthened review at the present day: its merits, which are many, and its defects, which are by no means inconsiderable, are as familiar to our readers as to ourselves; and therefore we shall "bestow none of our tediousness" in recapitulating either, but proceed at once to notice the peculiarities by which the present is distinguished from the former editions, and to enquire whether the work is so entirely changed as the announcement in the title-page would lead us to expect.

The fourth edition of Dr. Paris's treatise was published in 1830, and "it has remained out of print for a considerable period: so far, however, from this being a subject of regret, I rejoice," says the author, "at the opportunity it now affords me of collecting from the stores of a wider experience such facts as may tend to confirm and extend the doctrines, or to illustrate more clearly those general views which were submitted to the profession in the first edition." (P. 1.)

From these remarks, and from the numerous contributions which have been made to the physiological history of digestion since the publication of the preceding edition, seven years ago, we naturally took up the present volume with the expectation of finding it replete with those improvements which the progress of science demanded, and which the imposing assurance of being "nearly re-written" implied a consciousness on the part of the author of being due to his readers.

On comparing the present with the preceding edition, which we have taken some pains to do, we find it to contain rather less matter, and under a somewhat different arrangement. Instead of being divided into three parts as formerly, it is now divided into four, of which the second, "of Dietetic Observances founded on Physiological Principles," seems to have been suggested by the title of the second edition of Dr. Combe's work: but the novelty lies only in the title; for almost all the contents of that section appeared verbatim in the fourth edition, under different heads. Several pages of old matter have been entirely suppressed, and fifteen or twenty new paragraphs, amounting in all to about as many pages, have been introduced; but very few new facts or views have been contributed from the stores of experience, to which the author refers in the above quotation; and even those which are new have been either simply thrown into the heap or turned to very little account in any subsequent application to practice. In all other respects, then, the fifth is truly a transcript of the fourth edition, with all its excellencies, defects, and omissions.

It is with pain that we feel ourselves compelled to make such remarks on the writings of one who has been neither a slothful nor an useless labourer in the field of science. Had the volume been announced with less pretensions, and simply as a new and improved edition of a work already known, it would never have elicited from us a single disparaging line; but, when it is put forth in the guise of an almost new work, we are bound to put our readers on their guard against purchasing it as such; and, if the truth be disagreeable to any one, the fault lies not with us, but with the author himself, from whom alone the announcement could emanate.

Even where Dr. Paris has made any alteration on the text of his work, we are not aware that he has taken the opportunity of correcting any of his former errors, or of supplying any considerable omissions. Under the head of the anatomy of the digestive organs, for example, two meager paragraphs, about the functions of the nerves of the stomach, are added, in the first of which the author merely remarks, that, "*notwithstanding* the important discoveries of Sir C. Bell, there still remains some obscurity with respect to the influence of each particular nerve on the function of the stomach;" and forthwith dismisses the subject with a few vague generalities. Here, however, a twofold injustice is committed. Not only are the researches of Tiedemann, Gmelin, Philip, Magendie, and, above all, Brâchet, passed over without an allusion, although they are the most interesting which have yet appeared on the special functions of the stomachic nerves; but, from the words quoted, the reader is led to consider Sir Charles Bell as the chief, if not the only, enquirer who has thrown any light on that branch of the subject; whereas, he has, in reality, done nothing to elucidate it, and has never entered very minutely into its investigation.

Another remarkable omission occurs when discussing the venous origin of the biliary secretion. Dr. Paris re-produces, *literatim*, the old, and comparatively unsatisfactory, evidence of Saunders, Simon, Abernethy, and Magendie; but never even hints at the existence of the far more conclusive experiments and researches of Kiernan, although the latter have now been some years before the public; and, again, when enquiring into the absorbing or non-absorbing power of the skin, as connected with nutrition, he justly declares the question to be one of great importance, but, nevertheless, does not add one word to his former imperfect account of it; although Edwards and others have since established the affirmative by direct experiment. Nay, even in discussing one of the principal topics of his own book,—Digestion, and its agent, the gastric juice,—he reiterates his former descriptions to the very letter, even to the quoting of Montegre's mistaken conclusions on artificial digestion; and altogether disregards the experiments and contributions of Beaumont, Brâchet, Schwann, Müller, and Tiedemann, which are not only the most recent but by far the most important and instructive which we possess.

The author's neglect of recent information, and literal adherence to his former text, are further exemplified in his very slender notice of the curious experiments performed by Dr. Beaumont, on the Canadian voyageur, Alexis St. Martin; of which we gave an account in a former Number, and which are now familiar to the profession. We consider this omission as blameable in an author writing *ex professo* on the subject to which they relate, because, without by any means adopting all Dr. Beaumont's results, we cannot be blind to the fact that the zeal and ability with which he availed himself of his singularly favorable opportunities, constitute a strong claim on the respect and attention, and even on the justice, of every succeeding enquirer. Dr. Paris cannot plead ignorance as an excuse; for, in one of his earliest pages, he refers to the confirmation of former results by the case of St. Martin as "remarkably striking;" and after quoting, from Dr. Combe's work, "the most interesting facts that were thus ascertained," he promises again "to advert to the observations of Dr. Beaumont;" but, except in the form of a mere allusion, he never does so.

It is not because Dr. Beaumont has made any important *discoveries* regarding digestion that we find fault with Dr. Paris's neglect of his labours. The chief value of his experiments, as Dr. Paris himself justly remarks, consists in the confirmation which they afford of known but hitherto uncertain facts, and in their substituting certainty for doubt where certainty is of great practical value. From the nature of the case, Dr. Beaumont was able to free his researches from many sources of error inseparable from experiments made on animals, or during bad health; and hence he has succeeded in adducing evidence on some points of a much more conclusive kind than any quoted by Dr. Paris; and in so far, consequently, the latter has done less justice to his subject than his readers had a right to require from him.

An amusing inconsistency appears in reference to the very first "fact" which Dr. Paris adopts as ascertained by Dr. Beaumont's experiments. After explicitly concurring with Dr. B. in stating that "gastric juice *is never found free in the stomach,* but is always excited to discharge itself by the introduction of food or some other irritant," Dr. Paris very quietly repeats, from his old edition, that one of the reasons why appetite is *not always felt* on rising in the morning is, "*because gastric juice may not be secreted in any quantity during the night*, while the muscular energies of the stomach, although invigorated by repose, are not immediately called into action," (p. 139;) just as if the accumulation of gastric juice in the stomach, in the absence of food, was one of the efficient causes of appetite, and had not been previously declared by himself never to occur!

Another contradiction, which existed in the former edition, and which recurs uncorrected in this, is where he first tells us, at page 35, that "*six or seven grains* of the inner coat of the stomach, *infused in water*, gave *a liquor* which *coagulated* more than *a hundred ounces* of milk;" and then, farther down on the same page, very gravely adds, "the *coagulating* and efficient principle, whatever it may be, is *evidently not diffusible* in that liquid." If the latter be true, what, in the name of wonder, is the liquor which is stated to coagulate the milk? According to our view, the experiment in the first sentence proves the coagulating principle to be *extremely diffusible* in water; and we cannot imagine what Dr. Paris can mean by affirming the contrary. We are induced to notice this contradiction more seriously than would otherwise have been necessary, because he has linked his erroneous inference with an important practical conclusion, shewing "how unfounded that opinion is which attributes to the potation of water the mischief of diluting the gastric juice, and thus of weakening the digestive process." Now if, as we have seen it to be, the gastric juice is really diffusible in water, the fact of its being diluted by such diffusion is surely the reverse of "unfounded." If, indeed, the author means merely that drinking water some time *before* a meal does not dilute the gastric juice, and thereby injure digestion, we entirely agree with him; but then the non-dilution in such circumstances proceeds, not from any repugnance in the one to mix with or be diffused in the other, but simply from the fact that there is then no gastric juice in the stomach for the water to mix with, as its secretion begins only with the ingestion of food: and, accordingly, experience shews that the copious potation of water soon *after* eating does injure digestion; and we think it probable that it so acts by then diluting the gastric juice secreted in consequence of the contact of food.

It has often struck us that writers on diet attend too little to the condition of the system at the time of eating, as an element in determining the kind and quantity of food which ought to be consumed; as the latter ought manifestly to vary according to the expenditure attending the age, sex, mode of life, and other circumstances. In Dr. Combe's work this principle was strongly enforced, as lying at the very foundation of all dietetic regulations; and, as we consider it to be of great practical importance, we are glad that it has been adopted by Dr. Paris.

"If there be one law," he says, "in the animal economy which, above every other, is irresistibly forced upon our attention, and which must command our unqualified dissent, it is this, that THE ADJUSTMENT OF SUPPLIES SHOULD ALWAYS BEAR A DIRECT RELATION TO THE WASTE AND GROWTH OF THE INDIVIDUAL; and yet it would be difficult to point out a principle so little regarded and so generally abused. Do we not daily see the adult indulging in an excess adapted only to the demands of a rapid growth? the sedentary and indolent feeding like the active and laborious?" &c. (P. 69.)

We confess, however, that, after seeing the principle thus prominently brought forward at the outset of the new edition, we felt considerable disappointment on finding its applications to practice overlooked by few so much as by Dr. Paris himself.

One of the few additions of any interest in the volume before us occurs at page 91, where Dr. Paris gives a succinct account of the ingenious views of Dr. Schultz, of Berlin, regarding the *cæcum* being the seat of a second digestion, especially of vegetable food. Most of our readers are aware that, in some cases of artificial anus occurring near the termination of the small intestines, Dupuytren often observed vegetables pass out almost unchanged, while no trace of the animal food eaten along with them could be perceived. He thence inferred that the digestion of vegetable substances was completed in the large intestines; and the accuracy of his conclusions was to a great extent confirmed by the subsequent experiments of Dr. Beaumont, and by the fact of the unusual development of the colon in vegetable feeders, and its comparative smallness in carnivorous animals. Dr. Schultz, however, goes a step further, and maintains that the digestion of vegetable matter is completed in the cæcum; that it is there once more acidified, and that the office of the cæcal valve is to prevent its premature passage into the colon. Viridet affirms, in support of this view, that, in rabbits, he has found the food become again distinctly acid in the cæcum, after having been neutralized by the bile in the small intestines. We think this not unlikely to be correct; but must wait for further confirmation, before receiving Dr. Schultz's views as established.

Most of the other alterations in the present edition occur under the head of Indigestion, but they are neither numerous nor important. The most deserving of attention on the part of the practitioner is, perhaps, the description which Dr. Paris gives of a peculiar appearance of the tongue, consisting of "a dirty brown fur, principally occupying one-half of the tongue, longitudinally, from its root to its tip," which he believes "may be very generally received as evidence of cerebral disturbance. In paralysis it is frequently very striking; but, in morbid states of the nervous system, short of such mischief, I have noticed its presence in a less defined form, and have found the conclusion deduced from it subsequently confirmed by other symptoms." (P. 342.) We strongly recommend this to the verification of our readers, and are glad to have the means of aiding Dr. Paris in calling the attention of the profession to its occurrence. The remarks on the examination of the intestinal discharges are also very good.

While we have thus freely criticised the imperfections and omissions of the present edition of Dr. Paris's work, we must not be considered as on that account condemning it as a whole: on the contrary, we recommend it to the student as containing a great deal of useful information. From the announcement of its being nearly re-written, we were certainly entitled to expect a higher degree of improvement in its pages than they really present; but, notwithstanding this defect, it contains much that is both interesting and practically useful.

VII. The announcement of Mr. Curtis's book, and our knowledge of some of his previous writings, did not, we confess, prepare us to see a work of the character of that now before us proceeding from his hand. Knowing it to be impossible that the author of several of the volumes that have appeared under his name could compose an original work worth reading on the subject of the present, we were not even disposed to believe that he could compile such a work from the writings of others. We must admit, however, (if he is the author of the volume before us,) that he has really succeeded in doing so. How Dr. Combe may be pleased to find such wholesale use made of his admirable treatise we know not; but we think it probable that his benevolent feelings may be gratified to see many of the valuable truths contained in it diffused through a new class of readers in the pages of Mr. Curtis. For our own parts, we pass over without comment the incongruity of a frontispiece representing Mr. West and his family, copied from West's picture; and allusions to the author's self and family; and a Greek motto; and the mention of ear-inventions and eye-inventions; and advertisements of countless publications, and declarations of loyalty to George III., and other peculiarities, in consideration of the cheapness of the work, its small size, and, generally speaking, its sound sense. It is but justice to the author to admit that there are some few observations in it that are either new or put in a new light. Whether a good or a bad aurist and oculist, Mr. Curtis must be allowed to have had no small experience of mankind.

In the observations on the skin, and on respiration, (chiefly taken as usual from Dr. Combe,) the impropriety of closing bed-curtains is enforced by stating that "it is a remarkable fact that if a canary-bird be hung up in a cage at night at the head of a bed with close-drawn curtains, it will be found dead in the morning." (P. 22.)

We trust that some of Mr. Curtis's readers may be impressed with the undeniable truth which he advocates, and which parents too seldom remember, that "the first years of life should be directed to laying the foundations of health, which are the foundations of happiness." Throughout all plans of education it is, indeed, deeply to be regretted, that views of worldly prosperity, (to a certain extent no doubt commendable, and even necessary to many kinds of virtue), predominate over considerations which affect the character of the person to be educated.

We are always glad to meet with proofs that the health of the poorer classes is thought worthy of especial attention. Mr. Curtis mentions the baths in the City Road, at Lambeth, and Brighton, which are thrown open to the public at such low rates as to exclude few from the comfort of bathing. The subject is well worthy of the attention of medical practitioners in all towns of sufficient size to support such establishments.

Among several miscellaneous remarks, we find one on the subject of sending young persons to the continent to be educated; which Mr. Curtis thinks is done at the expense, in many instances, of health and happiness. There are, we hope, many exceptions to this observation; but that much evil is often incurred may readily be supposed; for several of the young persons sent abroad for education are the children of indifferently educated people, whose chief desire is that their sons and daughters should talk French. We entirely agree with Mr. Curtis in condensing the toil of Sunday observances so common in schools. Early morning prayers, repetitions of collects; morning sermon; afternoon lecture; serious readings; evening lecture again, and evening prayers, concur in many establishments to make Sunday no day of rest. On habits of early rising we need make no remark. The example of the royal family, adduced by Mr. Curtis, is not a very happy one. The poor king, (William IV.) whose early training Mr. Curtis thought bid fair to make him "exceed in length of life any of the previous kings of England," died a month after the publication of this loyal prophecy!

The remarks on Diet are almost entirely taken from Dr. Combe. When making reference to Temperance Societies, Mr. Curtis quotes from Grund's work on America the statement that, in 1833, the American Temperance Society consisted of two millions of members; and that two years before, when the number was much less, 1500 distilleries had been stopped, 1000 vessels sailed from American ports without ardent spirits as a part of their provision, and 4,500 drunkards had been reclaimed. He mentions, also, that Mr. Livesey, of Preston, informs him that upwards of thirty thousand persons, chiefly mechanics and artisans, have joined the temperance societies in Lancashire. We have always considered these societies as temporary expedients, not to be despised; but to be superseded by a general up-raising of the moral and intellectual condition of the working classes.

It is truly observed that there are many young men in the city who are engaged in sedentary occupations all the day, and yet, by riding to and from their offices in omnibuses, deprive themselves of the only opportunity they possess of taking exercise. In this respect Mr. Curtis thinks their elder brethren are wiser; and he recommends them to live two or three miles out of town, and to consider a plot of garden ground as an indispensable appendage to their dwellings; so that, becoming early risers, they may cultivate their gardens and improve their complexions. Some useful remarks are made on moderating the passions which actuate the busy, the ambitious, and the sentimental; and at page 76, Mr. Curtis favours us with a description of the symptoms of love, or of the disease of love in excess; and bad enough they seem to be:—

"The progress of the disease of which excessive love is productive, may be thus described: as the force of love prevails, sighs grow deeper, a tremor affects the heart and pulse, the countenance is alternately pale or red, the voice is suppressed in the fauces, the eyes grow dim, cold sweats break out, sleep absents itself, at least until the morning, the secretions become disturbed, and a loss of appetite, a hectic fever, melancholy, or perhaps madness, if not death, constitute the sad catastrophe." (P. 76.)

Mr. Curtis disputes an opinion of Mr. Mayo, given in the Philosophy of Living, that "the best instrument for conversation with a single person is the ear-trumpet with an elastic tube;" and he strongly recommends his own "acoustic chair," the person sitting in which "hears at the opposite side from that at which he is addressed." (P. 105.) He pronounces Mr. Mayo equally unfortunate in eulogising his (Mr. M.'s) invention of grey glass spectacles "of a pure black diluted;" and decidedly prefers his own "wire-gauze" ones, made convex; which have "received the approbation of his majesty, who has ordered two pairs; and of the Emperor of Russia, who has purchased three dozen pairs, and constantly uses them in his journeys over the plains of snow." (P. 107.) The only serious objection, he says, that he has heard against them is that little profit attends the sale of them. Reflecting on these contrary opinions, we cannot consider that the public should be perplexed by the differences of doctors.

VIII. The work of M. Bureaud-Riofrey is on a less comprehensive subject than several of those already noticed. The subject, however, is of the very first importance. In an eloquent introduction, the author observes, that since the publication of Rousseau's Emile the physical education of women has been a subject treated with disdain, whereas the perfecting of the human race, he goes on to say, must commence by the physical perfection of women. He particularly regrets that of the treatises already written on the subject of woman, some are too learned to be read by the mothers of families, and others treat of subjects which can only be properly brought before medical readers. The public, thus left to themselves concerning the management of young women, have, he remarks, fallen into the hands of rhetoricians and fencing masters, who have applied the robust gymnastics of Sparta to the delicate women of modern cities. Their practices were indiscriminate, and they paid little attention, or none, to the influence of physical agents on the body, to the laws of life, the phenomena of growth, the distinction of constitutions, age, and customs. A new science has arisen, the orthopœdic, of which the object is to prevent or correct the faults of conformation, chiefly of the muscles and bones, and mostly by mechanical means, resolvable into extension and forced repose. M. Bureaud-Riofrey, whose work is dated London, and who describes his practice as principally confined to the diseases of women, has very laudably, therefore, employed himself in preparing a work adapted to female readers, enforcing attention to those elements of physical education the study of which may not only prevent defects of osseous and muscular organization, but control with salutary effects the development of every organ which is subjected to the influence of education, or of the will. Like most works of this kind addressed to popular readers, it has not a few pages of extraneous matter, introductory and collateral; but the practical remarks which it contains are indicative of observation and judgment.

In proceeding to treat of the influence of external agents he first notices Air, Caloric, and Light. This last forms the subject of a distinct chapter. It is difficult to convince mothers of the importance of this agent, although perhaps not more difficult than to impress them with a sense of the importance of good air, to the health of their children. Dr. Milne Edwards's experiments on the development of tadpoles in the dark and in light are alluded to. An animal which in the first stage of its existence has the characters of a fish,—a tail, branchi, and no limbs; and in the second stage no character of the fish, no tail, no branchi, and four limbs,—afforded an excellent subject for these experiments; and it is well known that the results proved that these changes, if not absolutely prevented, were singularly retarded by the privation of light. The infrequency of deformity among the Chaymas, the Caribs, the Mexicans, and Peruvians, is ascribed by M. Humboldt (we think on very insufficient grounds,) to the free action of light over their bodies, rather than to any peculiarities by which their life is distinguished from that of the inhabitants of more civilized countries; and it is certainly probable that the use of what the French call ***insolation***, or free exposure of the body to the sun and the air, might, as suggested by Dr. Edwards, be advantageously had recourse to in scrofulous affections and other maladies of degeneration. It is presumed by some physiologists that the frequent and free exposure of the body to the atmospheric air, in the fine climate of Greece, tended to the development of the exquisite forms which yet live in ancient works of art. The traveller in Ireland, if acquainted with the condition of the poor in our large manufacturing towns, may usefully compare appearances resulting on the one hand from habitual residence in dark habitations, and on the other from almost constant exposure to sunshine and air. The manufacturer's younger children leave the cellar or the garret, but only for the narrow court or damp neglected back street; but the Irish cottager's child, although born in even greater poverty, runs about, as soon as it can run at all, in the open air of a temperate climate, not at all overburthened with clothing. The pale, unhealthy, scrofulous character of the manufacturer's child is too well known; whereas it is impossible to imagine finer examples of childish beauty and grace than are beheld at the doors of the Irish cabins.

M. Bureaud-Riofrey attaches importance to the chapters on hereditary and acquired constitutions, in which, however, there is little that is new. He refers to M. Pariset, who says, in his energetic manner, " Give me the roughest peasant, accustomed to the severity of the seasons, and I will make him delicate, nervous, susceptible, one whose corpulence, whose fresh-looking yet pasty countenance, (*visage fleuri, empaté*,) shall announce how his fibre is weak and distended, and the cellular tissue largely drenched with fluids. Give me, on the contrary, a slender citizen, pale-faced, of weak organization, soft contexture, whose body wants energy, whose mind is timid, and I will make him a soldier, a hunter, a sturdy sailor, whose tenacious fibre, compact bones, dense muscles, and iron arm, shall constitute a man intrepid in danger!" M. Pariset quotes the example of a certain Abbé Rucelleri, whose delicacy in all matters was excessive. His only drink was water, fetched from a distance. The sun, a calm, the least excess of heat or cold, affected his constitution. The very fear of being ill obliged him to take to his bed. The very idea of vapours, (we still quote from M. Pariset,) originated with this abbé, a malady without malady, which finds occupation for the indolent and profits those who 'treat it: oppressed by trifles, he shrunk from every form of fatigue and trouble: at length, spurred on by ambition, or by revenge, he entered the service of Catherine de Medicis; and overcoming his horror of labour, he became so robust and active, that his friends, seeing him at work all day long, and scorning repose at night, riding post on indifferent horses, and eating and drinking whatever came before him, asked him if he knew anything of the Abbé Rucelleri, saying they could not imagine what had become of him, or who had taken his place, or into what other body his soul had migrated.—The chief use of such examples is the encouragement they give, even in cases of strong hereditary predisposition, to persevere in that form of physical education which thus promises to overcome it.

In the chapters on Growth and Alimentation we observe many excellent observations; and these are followed by several chapters devoted to subjects connected with the exercise of the body, the importance of which fully justifies the author in devoting so much space to it. It would, indeed, be difficult to exaggerate the value of that which appears to determine blood, nutrition, nervous energy, vitality, into any organ, or into all, according to its application; and which must consequently when neglected or attended to, materially influence the development of the body and of the mind in the years of growth. Erroneous notions on the subject of female beauty still lead to customs subversive alike of natural beauty and of health. Even the calisthenic exercises, which now may be said to form a regular part of female education, cannot avert the evils arising from the stiff and imprisoning corset: and, managed as they are in many schools, these very exercises are productive of as much evil as good, if not more. It can scarcely be doubted that the more violent exercises, the forced positions, and unnatural and ungraceful attitudes, are in every way unfitted to the female organization. A lover does not admire the hard muscles of a pugilist, nor masculine carriage and demeanour, but the soft and rounded forms natural to the female figure. A rational man does not desire that his wife should be a rope-dancer, a balancer, a climber of rigging, or a performer of summersets over a bar, but a mother. Neither can the finishing school do better for him: for its aims are uniformity and insipidity, and an apathetic mind; and he requires a friend and a companion.

M. Bureaud-Riofrey does not carry his admiration of gymnastic measures to the excess of considering them in every case sufficient for the cure of muscular imperfections, which, he justly observes, require also the use of tonics, good diet, salubrious air, baths, frictions, shampooing, &c., which the physician must prescribe. His general principle is, that the weakest muscles ought, in cases of inequality of strength in the two sides, to be the most exercised. Several methods of doing this are mentioned. We must confess a degree of apprehension concerning some of these, such as balancing vases or boards on the head, at least in the way practised in some schools, in which young ladies while occupied in drawing or music are required to balance a board upon their heads. The object in these cases is to produce that uprightness and even stiffness of carriage which is after all unsightly; whilst the torment of the means employed is in some irritable constitutions very serious, and the inconvenience must always much impede the progress of the pupil in what it is professed to teach her. If we do not go so far as to say that we have seldom seen unequal developments of muscles, once formed, entirely removed or manifestly checked by any means employed; we have at least often witnessed evil consequences from forcible efforts to depress shoulders, to flatten prominent chests, and to keep heads erect. But the prevention of such deformities is another matter, and may reasonably be hoped from the employment of a judicious hygiène.

If it can be justly said that the perfect development of any one part of the body is more important than that of the rest, it must be said of that of the chest. The best organized brain must even possess a limited range of exercise if there is not an ample chest in which the blood may receive the full benefit of the atmospheric air, and the nutritious fluid commingled with it undergo perfect assimilation. Unfortunately, as is too well known, this is the part of the body which is subjected to the most pernicious constraint, or if the same constraint is extended, it is to the epigastrium and hypochondria, where the most important organs of digestion are situated. We suspect M. Bureaud-Riofrey of a wish to appear singular when we find him defending the use of stays, and denying their bad effects. In a previous chapter he counsels their being disused during gymnastic exercises; yet he considers that they are useful to weak persons on the principle of a laced stocking. The same argument might be employed to recommend the universal use of laced stockings. It is surely inconsistent to recommend them as strengthening the muscles, and yet to discard them during exercises which call for more than usual exertions of muscular strength.

That which gives peculiar interest to the hygiène of young women is, that the observance or neglect of its rules may have a serious influence upon them at the age of puberty, when certain organs peculiar to the system of the female, and on the healthy development of which all her future health and comfort may be said to depend, are called, as it were, into existence. The general effort observable at this period in the female constitution, the uncertain direction of this effort, and the importance of its right direction, render everything in the management of a young person material. In great cities, M. Bureaud-Riofrey observes, the young woman arrives at puberty by the mind, by the imagination and the desires of the heart, long before actual puberty has taken place, and hence arise the disorder and struggle between the sensibility and the organization, and all those peculiarities which make this period of life, in the higher ranks, a period of disorder and suffering. Nothing, he adds, making use of the expressions of M. Georget, nothing is spared to obtain for them the fatal gift of sensibility; inactivity of the muscular system, the cultivation of music, society, balls, plays, mental indolence, books which excite the feelings, and give visionary pictures of life. We must remark, however, that whatever evils this management may produce, sensibility has little to do with producing them, although selfishness, which is the only feeling really nursed by such a system, may have a great deal to do with them. Such sensitive young ladies perform the finest compositions without understanding them, mix in society without powers of conversation, dance without enjoyment, see plays without feeling the charms of poetry or of acting, and read almost without amusement. And thus it results, that notwithstanding the spread of education, the diffusion of knowledge, and all that excites the enthusiasm of some and the fears of others, nothing is more rare than an intellectual young woman.

M. Bureaud-Riofrey has described the dangers, physical, moral, and mental, of the period of puberty in glowing language, but with strict truth, making some allowance for the nation in which his observation has probably been the most exercised. The pages he has devoted to this part of his subject, and the short chapter on beauty, in which he claims for medicine, with Descartes, the prospective honour of contributing largely to perfecting the human race, will, we hope, recommend his hygiènic precepts to many who would be insensible to higher arguments. And although the medical reader will find his book verbose, and often too diffuse for professional reading, he will not fail to meet in the course of the author's remarks on the phenomena of growth and puberty, and on the care required to conduct these phenomena successfully, many acute and valuable observations, indicative of a reflecting and philosophical mind.

IX. Dr. Caleb Ticknor's work, (whose title, by the way, Mr. Mayo has laid violent hands on,) has greatly interested us. Its general subject is, to be sure, only that of the works we have already had to notice; but its application to the citizens of the United States, and the glimpses it affords of the manners and mind of a people considered to enjoy a fuller share of free agency than is allowed or avowed in the older countries of the earth, impart to it a variety which makes us forget the sameness of the details to which we are accustomed in writings of this kind. Its very commencement is exciting.

"The author can assign no other cause for preparing this volume than the presence and universal prevalence in this country of a malady, an epidemic, the like of which was never before witnessed, sparing neither age, sex, nor condition, and being followed by the most unhappy consequences. If the reader ask the name of this disease, he may be told that it is a sort of mania, fanaticism, or ultraism; if he ask where, and in what it may be seen, he may be answered, in all places and in all things. It is seen in most of the charitable and benevolent operations of the day; in religious zeal, political warfare, morality and immorality; in most of the domestic concerns of life, and, in fact, in all the particulars and minutiæ of living, moving, and being. There seems a remarkable propensity in us Americans to run into unwarrantable extravagances: whatever scheme is adopted, or whatever plan devised, whether for good or evil, is carried to an extreme. To one who contemplates the present condition of our country with calmness and deliberation, everything would seem to be upside down, or in a state of the most perfect confusion. He would see men running into opposite extremes on all subjects, and man warring to the death with his brother or neighbour on some trivial question, while they are no better agreed on matters of the greatest moment. To judge of men by their actions, one would suppose that a great proportion were mad, and that the world was one immense madhouse. Retrenchment and self-mortification seem to be the order of the day in relation to food and drink; there being no virtue, on the principle of radicalism, which does not consist in going counter to the appetites and instincts of nature. 'Let us be temperate in our meats and drinks,' says one, 'and *use the world as not abusing it.*' 'No,' says another; 'but let us rather *eat no meat while the world stands;* and, as to drink, let that be cold water.' Such sentiments have been put forward on the subject of diet, and such ultra measures urged, that the very injury is caused which is attempted to be averted, —to wit, ill health and consequent unhappiness.

"The mind cannot thus continue in a feverish state of excitement; but, agreeably to the operations of nature's laws, it must pass into a diametrically opposite state,—into a state of depression proportioned to the previous excitement. We may, therefore, confidently anticipate a reaction; and, without some signal interposition, I verily believe that, within ten years, infidelity and apathy will bear a more triumphant sway in this country than has ever yet been known; and far less will be done in the benevolent operations which characterize this day than if our zeal had been tempered with more discretion." (*Pref.*)

Although the whole of Dr. Ticknor's volume is written with great liveliness, and contains much matter for observation, we must confine our attention almost entirely to points presenting some peculiarity arising out of the author's national position. Thus, we should leave the chapter on Diet untouched, but for the necessity under which Dr. Ticknor finds himself of combating one of the prevalent fanaticisms of America, in the shape of an "anti-animal" system of living. One Solyman Browne did, it appears, in 1833, publish a poem in New York, whereunto certain notes were appended by "E. Parmely, dentist;" and these notes were of a "truly vegetable, antiphlogistic character," setting forth how he, the said Parmely, having suffered much from a gross and improper diet, reformed his living, excluded all animal food and strong drinks, and was restored to health. This, Dr. Ticknor remarks, was all very well. Mr. Parmely led a sedentary life, and the abstemious plan, although suitable to him, was not on that account fit for every man in New York. Nor should he vainly quote Dr. Lambe, and assert that "an adherence to the use of animal food is no more than a persistence in the gross customs of savage life, and evinces an insensibility to the progress of reason, and to the operation of intellectual improvement." Dr. Ticknor very ably refutes these fancies, by an appeal to Combe and various other writers, and, above all, to experience and observation; pointing out, by reference to numerous vegetable-feeding nations, how little the absence of beef benefits the "progress of reason." Wishing to look upon this question without prejudice, he observes that the Irishman living on potatoes, and the Scotchman living on oaten cake, are not far behind their English neighbours, who, even at the worst, use so much more animal food; and we do not doubt Dr. Ticknor's assertion that "the slaves in the Southern States, with their usual allowance of a peck of corn each week, enjoy better health than their masters, with all the luxury that wealth can procure." The fact seems to be, that life and strength *may* be supported, to a certain extent, in any climate, upon any kind of food; but that the structure of man, his instinctive tastes, and the necessities induced by various climates, are all opposed to a fanatical abstemiousness wherever mixed food can be easily obtained. We admire the stern good sense with which Dr. Ticknor puts aside the dreams of dyspeptic writers, who would make all the world live by rule; of the folly of which he gives some pleasant examples:

"A young man commences study: he enters college, studies hard; his professor lectures him into sixteen ounces of food each day; to walk half a mile, fifteen rods, and three paces, just forty-three minutes after each meal; and to sleep six hours, thirty-three minutes, and forty-two seconds, every night. The consequence is, that the student, for a short time, makes rapid progress in his studies, and gives promise of future eminence; but soon he becomes pale, thin, and debilitated: he has dyspepsy, and he is dieted yet more; he is now deprived of all solid food; slops and gruel are his only allowance; and he finally adds another to the catalogue of singular dispensations. This is no overwrought picture, but a common, and quite too common, occurrence.

"It is strange to see a man tormenting his friends, and making himself miserable, by his unceasing and unavailing complaints, and his overweening anxiety about what he puts into his dear stomach. To-day he must have a little bran-bread, to-morrow a soda biscuit with a little milk, next a little rice and sugar, then rice and molasses; one day he is filled with wind, another with acid, and very often with whims and nonsense." (P. 48.)

We might accumulate proofs such as these of our American brethren having exhibited a great precocity of refinement. They pamper or starve themselves as fashion dictates; reject the regular doctors for quacks, who administer steam, red pepper, lobelia, and other articles of incendiary practice; and, in short, promise fair to imitate all the fashionable follies of older communities. In what relates to the happiness of life, physical, moral, and intellectual, we shall see, as we proceed, that republican institutions have yet done little for them. The causes of this it is not for us to consider, but we do not think that they lie very deep.

The probity of bakers is not greater in transatlantic regions than in

London itself. "The New York bakers will not use the Western flour, because, although it contains more starch, the chief aliment of bread, and is consequently more nutritious, yet it contains less of gluten, and, consequently, the baker is not able to raise so large a loaf from a given quantity of flour." So they use one eighth of sour flour, to make the dough rise, and the people of New York eat bad bread. The bad loaf swells into saleable size, and suits the baker better; but it swells after it is eaten, and distends the citizens with gaseous dyspepsia. But a righteous man, one Graham, makes what he calls "the staff of life," which is only bran bread, or bread made of unbolted flour, of which the said Graham asserts himself the inventor; and Graham's staff of life is all the fashion. Let us see whether the chapter on Drinks, affords equal proofs of advancing civilization.

Combating the abstemious fanatics of his country so effectively on the ground of animal diet, we were surprised to find Dr. Ticknor maintaining that because all animals except man can preserve health by drinking water, therefore man should drink nothing better, at any time, in any climate, at any age, in any circumstances or mood of mind. So used to teach our venerated Gregory; stoutly maintaining that wine was no more necessary to a Christian than to a Mahometan, nor to a man than to a horse: but we demurred to this, even at the time, being previously imbued with the milder doctrines of the ancients.

Philosophically considered, there may be some necessary connexion between stimulant liquors and the other stimuli peculiar to a creature of the highest intelligence and sensibility. The degradation of reason by their abuse is no argument against their use. The stimulus of many of the tender and nobler affections is equally unknown to the lower animals, but man is not on this account to discard them as unnatural to him. Man is often their slave; but his reason was given to him to rescue him from such slavery, and also from the practice of intoxicating himself with ardent spirits. The abuse of spirits has, however, been carried to such a frightful extent in America, that we cannot be surprised to find several well-wishers of their country desirous of seeing their use utterly abolished. Dr. Ticknor subsequently acknowledges that a glass of wine may now and then be taken without doing any irreparable mischief: he evidently disapproves of the enthusiastic extremes of some of the temperance people; and, although he wishes "that every man were a *cold water* man," thinks that a moderate use of artificial stimuli may be permitted to old people. His good sense has an evident struggle with a favorite theory.

To those acquainted with the nauseous habits arising out of the custom of smoking, and of which almost all travellers in America have recorded their disgust, it will give pleasure, but no surprise, to learn that Dr. Ticknor devotes a chapter to The Great Pleasure and Benefit of Using Tobacco; in which after reciting its history, and relating instances of its poisonous effects, he censures with effective irony the customs of smoking, snuff-taking, and chewing, pointing out the insensibility to smell which these induce, the ruin of the voice, the deterioration of the breath, and of the manners. From these habits, and these consequences, even the ladies do not appear to be free.

"Could any one," he says, "entirely unacquainted with the unaccountable habits and propensities of man, after knowing the properties of tobacco, be made to believe that half the adult inhabitants of America are passionately addicted to its use? And were he told that America's fairest daughters use it too, would he not be perfectly incredulous? And were he told, further, that ladies of the greatest respectability, the most genteel and accomplished in one of our largest cities, carry their little jars or boxes of snuff into the social circle, and, with a delicate ivory spoon, feed their *sweet* mouths with this most delicious and agreeable poison, would he not be petrified with astonishment! How delightful it must be to have an amiable spouse rendered doubly *sweet*, and bewitchingly interesting, and most charmingly stupid and idiotic, by the constant practice of *chewing snuff;* and what a fine example to children, of the pure, cultivated taste and self-control of the mother!" (P. 112.)

On the subject of Dress we do not find that the fair Americans are in any degree behind or before Europeans. They lace with equal severity, and even with more system and perseverance than our countrywomen. By way of opposing this hurtful fashion, Dr. Ticknor is at much pains to teach the uses of respiration, and the impediment of that important function created by tight-lacing. He appeals to the Venus de Medicis, introduces the well-known outlines of her figure and that of a modern belle, both dressed and undressed, and tries to make an impression on the American ladies, whom, in Transatlantic fashion he calls *females,* by telling them that if they would only be quiet and lace not, a great proportion of them "would closely approximate, if not quite equal, the divine standard." He takes scarcely less pains to convince them that scanty or insufficient clothing is full of danger to the health. The case is evidently desperate; for, in obedience to fashion, the ladies "defy physical laws, breathe freely in armour, and are warm in gossamer."

"But a short time has elapsed since it was considered unfashionable, or ungenteel, to wear any other than cloth shoes, with a very thin single sole, even in the most severe winter weather. And what is very singular, no lady was ever known to complain of cold feet—they were *always warm, quite warm, not the least uncomfortable*: were we to give full credence to the devotees of fashion, we should never find health injured, or comfort abridged, by any custom, however rigid, or however absurd. The miss in her teens, her mother of forty, or her grandmother of sixty, though so tightly laced as to perform but half a respiration, will tell you, and insist upon it, they are so loosely dressed that their clothes will hardly stay on, and that they breathe with the most perfect freedom. A lady in midwinter, with open-worked stockings, and only a pair of thin cloth shoes, will most unhesitatingly say, that her feet are never cold: and if her neck is at the same time bare, will say that she feels no inconvenience, that she is indeed very warm." (P. 147.)

The chapter on the Management of Young Children teaches us that the Americans put a tight bandage round the bodies of infants, leave the arms of boys and girls bare, dress children of all ages very lightly, and dose them with paregoric. The practice of giving them stimulants is nearly obsolete; but Dr. Ticknor says, "not long since I met with an old fashioned mother, who wondered that her baby grew so very slowly and looked so puny, for she fed him with *gin-sling* two or three times every day." It seems, indeed, that until very recently, many very pious and good women thought it impossible to rear children without giving them milk-punch. Both mothers and nurses continue to ply children with food, with the admirable assiduity so observable in the nurseries of the mother country. He very justly observes that infants often cry from thirst, which neither food nor the breast will relieve, but which a little cold water will entirely allay.

We learn from the chapter on Amusements, that the theatres of America present a combination of the fashionable follies of the opera of London with the disgusting scenes exhibited at our third and fourth-rate theatres; and that the public taste seems rather to become more degraded than to improve. Indecent exhibitions, immoral sentiments, incorrect company, drinking, smoking, rude conversation, and late hours, are no less common to American theatrical amusements than to those of England, which in these respects are far more offensive than those of the continent. Nor have the Americans the good sense to begin dancing at their balls before the hour of sleep; whilst the ladies, on such occasions more tightly laced than ever, sometimes expire in the midst of a dance.

These follies are fully accounted for in the chapter on Education; by which we learn that the young are confined for many hours to all the old monotonies of unreformed schools, now almost unknown in England; sit on hard and backless benches for six hours out of twenty-four; and whilst the boys are urged and stimulated to all acquisitions which can subserve the purposes of ambition or of mammon, the girls conclude what is called their education at seventeen, and sit down, frivolous, and fine, and uncompanionable for life. The consequences may be plainly seen, as depicted in the works of Dr. Brigham and of Dr. Ticknor. An ill-regulated, because excessive, pursuit of worldly objects, associated with a fanatical and exciting religion, exhibits its effects on the minds of the American population to a very fearful extent. We trust the writings of the several able physicians of that great nation, who have directed their attention to the subject of hygiène, will awaken the energetic communities in which they dwell to their truest interest and highest wisdom; and that among the proud distinctions of a country, to which no friend of mankind can look without the deepest interest, will soon be that of having carried into practical effect the Philosophy of Living.

It was not our intention in the foregoing pages to give a regular account of any of the works we have noticed; which would have required more space than could with propriety have been allotted to a subject not new to medical men, and chiefly concerning unprofessional persons: but we did not think it proper to pass by without notice publications of which the general object was excellent, (for such may be said of all,) and which we believe calculated to do essential public service. The medical observer, viewing the errors of mankind without passion, and knowing how many are inherited, how many inherent in organization, how many the result of unhappy circumstances, becomes a lenient judge of his fellow-creatures, and above all things characterized by his humanity. Well knowing that one of the first and most constant conditions for remedying or preventing these social and moral evils is to preserve the integrity of the physical frame, he can never view with indifference any attempts to instruct mankind concerning the means of preserving that perfect health without which, it may be said without hyperbole, they can neither be fully useful or morally good, or transmit good dispositions to those who are to come after them.

